# Supplementary material for: Non-Invasive Assessment of Skin Surface Proteins of Psoriasis Vulgaris Patients in Response to Biological Therapy
Source: Int J Mol Sci. 2023 Nov 13;24(22):16248. doi: 10.3390/ijms242216248 (PMC10671061; doi:10.3390/ijms242216248)
Supplement: Supplementary file 1 [file ijms-24-16248-s001.zip › ijms-2691541-supplementary.pdf]

Supplementary Materials

Content

Materials and Methods 2

Transdermal Analysis Patch 2

Composition of the Transdermal Analysis Patch 2

Commercial antibody Kits used for TAP 3

Participant characteristics 4

Characteristics of recruited psoriasis vulgaris patients 4

Supplementary Table S2. Characteristics of the recruited patients (N = 37) 4

Figures 8

Supplementary Figure S1. Changes in the psoriasis area severity index (PASI) and local scores of induration, desquamation, and erythema induced by biological therapy. 8

Materials and Methods

Transdermal Analysis Patch

Composition of the Transdermal Analysis Patch

Transdermal Analyses Patch (TAP; FibroTx LLc, Tallinn Estonia) consists of a micro-array that is supported by a dermal adhesive plaster (3M White Nonwoven Medical Tape, 9907HTW) for easy fixture to skin. In between the antibody microarray and the plaster, a layer is positioned that serves as a fluid reservoir for the buffer needed for protein capturing from the skin. In addition, this expandable layer serves as a pressure pad to ensure close contact of the microarray to the skin. Each TAP micro-array contains two spots of positive controls (IgG; Goat anti-human IgG Lab AS, Estonia) to determine the specificity of biomarker measurements, and a panel of capturing antibodies, of which each capturing antibody variant is printed in triplicate (N = 2), as discrete spots on the membrane (GE HealthCare, 10600002) by non-contact dispensing (BioDot AD3400 printer). The concentrations of capturing antibodies (PeproTech EC Ltd, UK) used for TAP capturing antibody micro-arrays were as follows: 2.25 ng of anti-hBD-1, 0.5 ng of anti-hBD-2, 0.25 ng of anti-IL-1α, 0.75 ng of anti-CXCL- 1/2, and 0.6 ng of anti-CXCL-8 per spot, additionally each micro-array contained a negative control (PBS - with 20% (v/v) glycerol) and positive control (0.03 ng biotinylated anti- hBD-1). Captured proteins are analysed, both qualitatively and quantitatively, on the antibody micro-array using spot-ELISA.

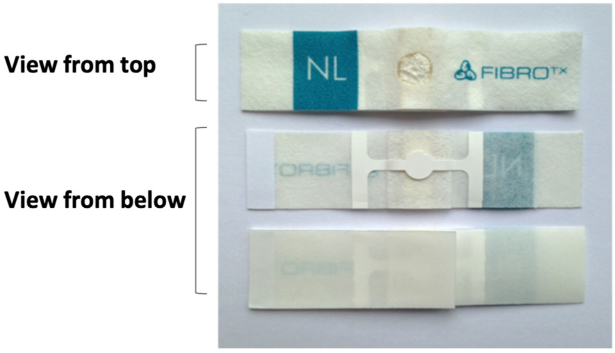

Figure S2. Composition of the Transdermal Analyses Patch (TAP). TAP consists of a plaster with a nitrocellulose core that contains the capture antibody microarray clearly visible in the middle.

Visualisation of captured proteins using spot-ELISA

48-well plates (Greiner BioOne) for assay were blocked with 1% BSA (w/v) (Capricorn Scientific, PN: BSA-1T) in PBS (pH = 7.4). Subsequently, wells were washed with milli-Q water and dried. For further processing TAP capture antibody micro-arrays were placed into blocked 48-well plates and wetted with PBS. To create standard curves, capture antibody microarrays were incubated for 20 minutes at 33°C with a mixture of recombinant proteins diluted in PBS + 0.05% (v/v) Tween-20 (AppliChem, A4974). Unbound proteins were washed from the membrane with a wash buffer.

To determine the concentrations of skin surface captured biomarkers antibody capturing micro-arrays incubated on the skin were removed from plaster and placed into blocked 48-well plates and washed using wash buffer. Antibody capturing micro-arrays subjected to standard curves and skin surface biomarker analysis were blocked for 20 min at room temperature in 5% BSA (w/v) in PBS (pH = 7.4). A biotinylated secondary antibody was added to each capturing antibody microarray and incubated for 45 min at room temperature. The Catalysed Signal Amplification (CSA) System (Dako, K-1497) was used for signal amplification. Tyramide and Anti-Fluorescein-HRP solution was diluted in diluent (PBS + 0.05% (v/v) Tween-20) to 10% and micro-arrays were incubated for 15 min at room temperature. For signal visualisation, Substrate-Chromogen solution diluted to 0.4% in Substrate Buffer Concentrate was used, and micro-arrays were incubated for 15 min at room temperature. The reaction was stopped with milli-Q water. Signals of captured biomarkers were quantified by comparing the signals of these proteins captured from the skin of subjects using FibroTx TAP capture antibody micro-array incubated with fixed amounts of recombinant proteins.

Commercial antibody Kits used for TAP

Human GRO-ß (CXCL-2) ELISA Development Kit (Cat. No: 900-K120, PeproTech EC Ltd, UK), Human IL-1α ELISA Development Kit (Cat. No: 900-K11, PeproTech EC Ltd, UK), Human hBD-1 ELISA Development Kit (Cat. No: 900-K202, PeproTech EC Ltd, UK), Human hBD-2 ELISA Development Kit (Cat. No: 900-K172, PeproTech EC Ltd, UK), Human CXCL-8 ELISA Development Kit (Cat. No: 900-K18, PeproTech EC Ltd, UK).

Participant characteristics

Characteristics of recruited psoriasis vulgaris patients

The patients enrolled as part of this study were unrelated Caucasians living in Estonia and were recruited from the outpatient clinic between 2016 – 2020 as they arrived for the appointment, with no discrimination with regard to the clinical characteristics of the disease. The mean ± standard deviation (SD) of the age of patients was 43.75 ± 11.37, respectively. Of 37 patients 27 were male and 10 were female (Supplementary Table S1).

Supplementary Table S1. Characteristics of the recruited patients (N = 37) before the treatment initiation

Sex	Age	PASI	Induration	Erythema	Desqua-mation	Applied treatment	Body area
F	28	11.6	2	2	2	Infliximab	buttock; right; dorsal
M	34	4.5	2	2	2	Infliximab	crus; left; lateral
M	52	18.3	2	3	2	Infliximab	lower back; right; dorsal
F	45	14.4	2	3	2	Infliximab	forearm; right; medial
M	59	28.6	2	3	2	Infliximab	thigh; right; lateral
M	53	8.4	2	3	2	Infliximab	trunk; side; left; lateral
F	58	10.9	2	3	2	Infliximab	arm; left; lateral
M	55	4.5	2	2	2	Adalimumab	side; right; lateral
M	41	10.1	2	2	2	Ustekinumab	forearm; left; ventral
F	21	11.8	1	1	1	Secukinumab	buttock; right; dorsal
M	40	4.3	1	1	1	Adalimumab	crus; left; medial
M	44	3.6	2	2	2	Ustekinumab	forearm; right; ventral
M	45	N/A	2	3	2	Ustekinumab	arm; left; lateral
M	34	7	2	2	2	Adalimumab	forearm; left; lateral
M	42	4.5	2	3	2	Adalimumab	arm; right; lateral
M	40	4.5	2	2	2	Ustekinumab	elbow; right; unknown
M	51	26.4	2	2	3	Adalimumab	arm; left; lateral
M	44	3.6	2	2	2	Adalimumab	hand; right; dorsal
M	60	8.7	2	2	2	Ustekinumab	forearm; left; lateral
F	62	30.9	2	2	3	Adalimumab	forearm; left; lateral
M	53	4.5	1	3	1	Secukinumab	forearm; right; lateral
M	26	2.3	1	2	1	Secukinumab	elbow; left; unknown
F	29	6.4	3	3	1	Adalimumab	right, dorsal; upper back
F	44	6.5	2	1	2	Adalimumab	side; right
M	37	5.3	2	2	1	Adalimumab	forearm; right; lateral
M	42	4.6	2	2	1	Ustekinumab	forearm; right; lateral
F	26	7.6	1	3	1	Adalimumab	arm; right; lateral
M	55	16.1	1	2	1	Adalimumab	arm; left; lateral
M	43	14.6	2	2	2	Ustekinumab	side; right; ventral
M	40	62.4	4	4	4	Infliximab	sternum; left
M	57	6.6	1	2	1	Adalimumab	elbow; right; lateral
M	58	19.8	1	3	2	Adalimumab	elbow; left; lateral
M	58	12.8	3	3	2	Adalimumab	thigh; left; dorsal
M	31	6.8	1	3	1	Secukinumab	back, right; dorsal
F	29	7.7	1	3	1	Secukinumab	abdomen; left; medial
F	50	4.6	1	1	1	Secukinumab	thigh; left; medial
M	33	13.2	2	2	2	Secukinumab	forearm; right; lateral

Supplementary Table S1. Characteristics of the recruited patients. Disease severity was quantified as Psoriasis Area and Severity Index (PASI) score and as local scores for erythema (0 – 4), induration (0 – 4), and desquamation (0 – 4). F- female; M – male; L -psoriatic lesion, N/A – not analysed.

Supplementary Table S2A. Measurements of hBD-1, hBD-2, IL-1α, CXCL-1/2, and CXCL-8 on lesional skin of treatment responsive psoriasis patients during anti-TNFα targeted biological therapy.

Time point	Baseline		T1		T2		T3		T4		T5	
Biomarker	ng/ml	± SD	ng/ml	± SD	ng/ml	± SD	ng/ml	± SD	ng/ml	± SD	ng/ml	± SD
hBD-1	0.22	0.29	0.24	0.30	0.10	0.11	0.22	0.33	0.08	0.07	0.11	0.16
hBD-2	4.31	2.35	4.23	2.30	3.48	2.43	1.60	1.99	0.88	1.19	0.70	1.12
IL-1α	0.14	0.29	0.32	0.43	0.74	0.77	0.27	0.32	1.18	1.17	1.30	1.06
CXCL-1/2	0.04	0.07	0.02	0.04	0.01	0.02	0.01	0.02	0.00	0.01	0.01	0.02
CXCL-8	0.27	0.43	0.11	0.21	0.03	0.10	0.002	0.01	0.01	0.02	0.07	0.24

Supplementary Table S2A. Measurements of hBD-1, hBD-2, IL-1α, CXCL-1/2, and CXCL-8 on lesional skin of treatment responsive psoriasis patients during anti-TNFα targeted biological therapy. Biomarker measurements were performed on psoriasis vulgaris patients (N=15) lesional skin before treatment T0 (baseline), and after treatment initiation on the following time points over therapy: T1(week 2), on T2 (week 4); on T3 (week 12), on T4 (week 24), T5 (week 32). The skin surface protein measurements were sampled always at the same skin site using FibroTx TAP.

Supplementary Table S2B. Changes in the psoriasis area severity index (PASI) and local scores for erythema, induration, and desquamation of treatment-responsive psoriasis patients induced by anti-TNFα targeted biological therapy.

Time point	Baseline		T1		T2		T3		T4		T5	
Biomarker	Average score	± SD	Average score	± SD	Average score	± SD	Average score	± SD	Average score	± SD	Average score	± SD
PASI	14.27	14.17	11.82	9.17	6.05	5.87	3.33	4.30	1.07	1.67	1.63	3.28
Erythema	2.61	0.70	2.12	0.49	1.22	0.73	0.72	0.83	0.22	0.43	0.33	0.49
Desquamation	1.94	0.73	1.65	0.61	0.94	0.54	0.56	0.98	0.28	0.46	0.22	0.43
Induration	2.06	0.73	1.76	0.56	1.00	0.59	0.44	0.78	0.22	0.43	0.22	0.55

Supplementary Table S2B. Changes in the psoriasis area severity index (PASI) and local scores for erythema, induration, and desquamation of treatment-responsive psoriasis patients induced by anti-TNFα targeted biological therapy. The clinical scores were assessed on psoriasis vulgaris patients (N = 15) lesional skin before treatment T0 (baseline), and after treatment initiation at the following time points over therapy: T1(week 2), on T2 (week 4); on T3 (week 12), on T4 (week 24), T5 (week 32) always at the same lesion sites. The range of PASI score is 0 – 72, scores of erythema, desquamation and induration is on 0 – 4 scale.

Supplementary Table S3A. Measurements of hBD-1, hBD-2, IL-1α, CXCL-1/2, and CXCL-8 on lesional skin of treatment responsive psoriasis patients during anti-IL-17A targeted biological therapy.

Time point	Baseline		T1		T2		T3		T5	
Biomarker	ng/ml	± SD	ng/ml	± SD	ng/ml	± SD	ng/ml	± SD	ng/ml	± SD
hBD-1	0.34	0.22	0.26	0.23	0.14	0.20	0.15	0.17	0.11	0.10
hBD-2	6.68	5.25	7.82	5.34	1.60	2.09	0.00	0.00	0.02	0.45
IL-1	0.18	0.31	0.38	0.49	1.37	0.78	1.12	1.78	1.40	0.69
CXCL-1/2	0.02	0.01	0.00	0.00	0.00	0.00	0.00	0.00	0.00	0.00
CXCL-8	0.42	0.27	0.15	0.30	0.25	0.23	0.04	0.08	0.00	0.00

Supplementary Table S3A. Measurements of hBD-1, hBD-2, IL-1α, CXCL-1/2, and CXCL-8 on lesional skin of treatment responsive psoriasis patients during anti-IL-17A targeted biological therapy. Biomarker measurements were performed on psoriasis vulgaris patients (N = 5) lesional skin before treatment T0 (baseline), and after treatment initiation on following time points over therapy: T1(week 2), on T2 (week 4); on T3 (week 16), T5 (week 32). The skin surface protein measurements were sampled always at the same skin site using FibroTx TAP.

Supplementary Table S3B. Changes in the psoriasis area severity index (PASI) and local scores for erythema, induration, and desquamation treatment responsive psoriasis patients induced by anti- IL-17A targeted biological therapy.

Time point	Baseline		T1		T2		T3		T5	
Biomarker	Average score	± SD	Average score	± SD	Average score	± SD	Average score	± SD	Average score	± SD
PASI	8.8	3.9	5.3	1.8	2.4	1.7	0.0	1.8	0.1	1.3
Erythema	2.0	1.0	1.8	0.5	1.2	0.4	0.0	0.0	0.0	0.0
Desquamation	1.2	0.4	1.0	0.0	0.4	0.5	0.0	0.0	0.0	0.0
Induration	1.2	0.4	1.0	0.0	0.6	0.5	0.0	0.0	0.0	0.0

Supplementary Table S3B. Changes in the psoriasis area severity index (PASI) and local scores for erythema, induration, and desquamation treatment responsive psoriasis patients induced by anti- IL-17A targeted biological therapy. The clinical scores were assessed on psoriasis vulgaris patients (N = 5) lesional skin before treatment T0 (baseline), and after treatment initiation on the following time points over therapy: T1(week 2), on T2 (week 4); on T3 (week 16), on T5 (week 32) always at the same lesion sites. The range of PASI score is 0 – 72, scores of erythema, desquamation and induration is on 0 – 4 scale.

Supplementary Table S4A. Measurements of hBD-1, hBD-2, IL-1α, CXCL-1/2, and CXCL-8 on lesional skin of treatment responsive psoriasis patients during anti-IL-12/23 targeted biological therapy.

Time point	Baseline		T2		T3		T4		T5		T6	
Biomarker	ng/ml	± SD	ng/ml	± SD	ng/ml	± SD	ng/ml	± SD	ng/ml	± SD	ng/ml	± SD
hBD-1	0.16	0.09	0.12	0.11	0.09	0.12	0.08	0.08	0.18	0.18	0.15	0.12
hBD-2	3.41	2.09	3.16	2.15	1.47	2.60	1.43	2.11	1.66	2.61	1.84	2.88
IL-1	0.12	0.20	0.59	0.50	1.11	1.32	0.70	0.46	1.29	1.67	1.58	1.56
CXCL-1/2	0.05	0.04	0.01	0.02	0.00	0.01	0.00	0.00	0.00	0.00	0.00	0.01
CXCL-8	0.15	0.16	0.00	0.01	0.03	0.04	0.07	0.13	0.03	0.05	0.08	0.20

Supplementary Table S4A. Measurements of hBD-1, hBD-2, IL-1α, CXCL-1/2, and CXCL-8 on lesional skin of treatment-responsive psoriasis patients during anti-IL-12/23 targeted biological therapy. Biomarker measurements were performed on psoriasis vulgaris patients (N = 7) lesional skin before treatment T0 (baseline), and after treatment initiation at following time points over therapy: T2 (week 4), on T2 (week 4); on T3 (week 16), T4 (week 28), T5 (week 40), T6 (week 52). The skin surface protein measurements were sampled always at the same skin site using FibroTx TAP.

Supplementary Table S4B. Changes in the psoriasis area severity index (PASI) and local scores for erythema, induration, and desquamation treatment responsive psoriasis patients induced by anti- IL-12/23 targeted biological therapy.

Time point	Baseline		T2		T3		T4		T5		T6	
Biomarker	Average score	± SD	Average score	± SD	Average score	± SD	Average score	± SD	Average score	± SD	Average score	± SD
PASI	7.68	4.27	4.13	1.71	0.94	0.91	1.13	1.42	0.47	0.69	0.56	0.74
Erythema	2.14	0.38	1.29	0.49	0.43	0.53	0.57	0.79	0.14	0.38	0.14	0.38
Desquamation	1.86	0.38	1.00	0.82	0.29	0.49	0.14	0.38	0.14	0.38	0.14	0.38
Induration	2.00	0.00	1.14	0.69	0.43	0.53	0.29	0.49	0.14	0.38	0.14	0.38

Supplementary Table S4B. Changes in the psoriasis area severity index (PASI) and local scores for erythema, induration, and desquamation treatment responsive psoriasis patients induced by anti-IL-12/23 targeted biological therapy. The clinical scores were assessed on psoriasis vulgaris patients (N=7) lesional skin before treatment T0 (baseline), and after treatment initiation on following time points over therapy: T2 (week 4), on T2 (week 4); on T3 (week 16), T4 (week 28), T5 (week 40), T6 (week 52), always at the same lesion sites. The range of PASI score is 0 – 72, scores of erythema, desquamation and induration is on 0 – 4 scale.

Figures

Supplementary Figure S2. Changes in the psoriasis area severity index (PASI) and local scores of induration, desquamation, and erythema induced by biological therapy.

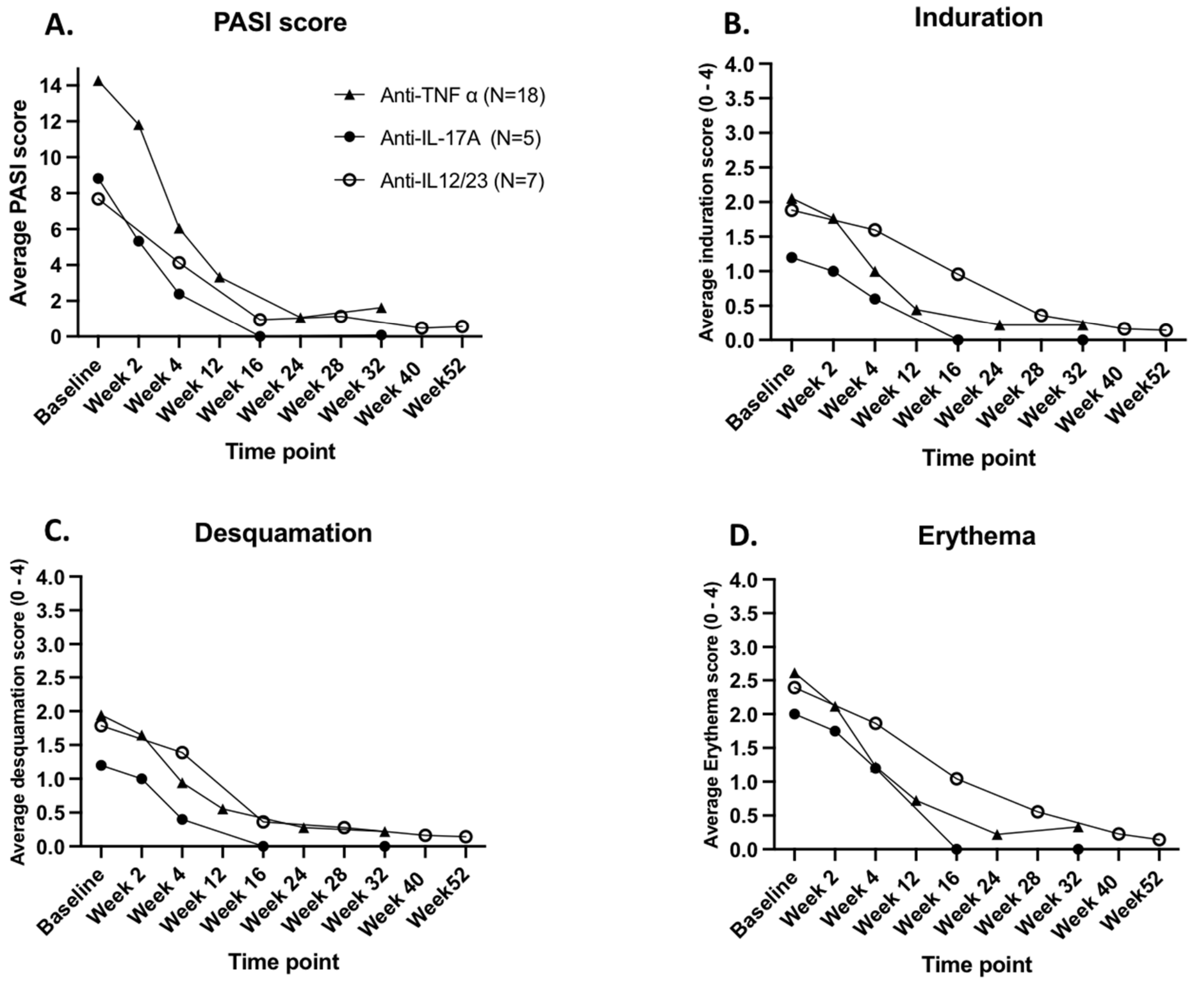

Figure S1. Changes in the psoriasis area severity index (PASI) and local scores of induration, desquamation, and erythema induced by biological therapy. The PASI (panel A), and local scores of Induration (panel B), Desquamation (panel C), and Erythema score (panel D) were documented before the treatment initiation at baseline and after treatment initiation on week 2- week 52, respectively over therapy at the same lesion sites. Each line plotted in Figure S1 represents a combined average measurement of a clinical score of all analysed psoriasis patients (black circles, N = 37), super responders (white squares, N = 20), responders (black triangles, N = 30), and non-responders (white triangles, N = 7) to the therapy. Y-axis: Average clinical score of analysed patients on the lesional skin in ng/ml, X-axis: sampling time point.
